# The Role of New Technologies in the Diagnosis and Surveillance of Non-Muscle Invasive Bladder Carcinoma: A Prospective, Double-Blinded, Monocentric Study of the XPERT© Bladder Cancer Monitor and Narrow Band Imaging© Cystoscopy

**DOI:** 10.3390/cancers14030618

**Published:** 2022-01-26

**Authors:** Gad Singer, Venkat M. Ramakrishnan, Uwe Rogel, Andreas Schötzau, Daniel Disteldorf, Philipp Maletzki, Jean-Pascal Adank, Marc Hofmann, Tilo Niemann, Sylvia Stadlmann, Antonio Nocito, Kurt Lehmann, Lukas J. Hefermehl

**Affiliations:** 1 Institute of Pathology, Kantonsspital Baden, CH-5404 Baden, Switzerland; gad.singer@ksb.ch (G.S.); uwe.rogel@ksb.ch (U.R.); 2 Division of Urology, Brigham and Women’s Hospital, Harvard Medical School, Boston, MA 02115, USA; venkatmr88@gmail.com (V.M.R.); sylvia.stadlmann@ksb.ch (S.S.); 3 Eudox Statistics, CH-4000 Basel, Switzerland; info@eudox.ch; 4 Department of Surgery, Division of Urology, Kantonsspital Baden, CH-5404 Baden, Switzerland; daniel.disteldorf@ksb.ch (D.D.); philipp.maletzki@ksb.ch (P.M.); jean-pascal.adank@ksb.ch (J.-P.A.); marc.hofmann@ksb.ch (M.H.); kurt.lehmann@ksb.ch (K.L.); 5 Department of Radiology, Kantonsspital Baden, CH-5404 Baden, Switzerland; tilo.niemann@ksb.ch; 6 Department of Surgery, Kantonsspital Baden, CH-5404 Baden, Switzerland; antonio.nocito@ksb.ch

**Keywords:** bladder carcinoma, urine, test, surveillance, biomarker, mRNA, narrow band imaging, XPERT

## Abstract

**Simple Summary:**

Patients with bladder cancer (BC) require close follow-up with white-light cystoscopy (WLC) and cytology. In this study, we sought to assess (a) the performance of a novel cystoscopy technology based on Narrow Band Imaging© (NBI), and (b) a new urine test (XPERT© Bladder Cancer Monitor, XBCM) that detects cancer proteins. We compared these to the established standard follow-up diagnostics. Our study showed that NBI cystoscopy does not provide any additional benefit over standard WLC. However, the XBCM urine test performed particularly well in instances of aggressive high-grade tumor recurrence. Therefore, XBCM may have enhanced utility in the early detection of potentially harmful BC recurrence.

**Abstract:**

Follow-up is essential for the early detection of recurrent non-muscle invasive bladder cancers (NMIBC). This study investigates the clinical relevance of new diagnostic tools such as an mRNA-based urine test (XPERT© Bladder Cancer Monitor, XBCM) and Narrow Band Imaging© (NBI) and compares them with the established follow-up diagnostics (white-light cystoscopy (WLC) and urine cytology). This was a prospective, double-blind, single-center study that involved patients undergoing NMIBC screening at a tertiary care center. Enrollment occurred between January 2018 and March 2020. In addition to standard care (WLC, cytology, and ultrasound), patients underwent XBCM urine testing and NBI cystoscopy. In total, 301 WLCs were performed; through this, 49 patients demonstrated NMIBC recurrence. NBI cystoscopy was congruent with WLC in all patients. Cytology showed a sensitivity (SE) and specificity (SP) of 27% and 97% (PPV: 65%; NPV 87%), respectively, whereas XBCM showed SE and SP of 58% and 89%, respectively (PPV: 51%; NPV: 92%; AUC: 0.79 (0.716–0.871)). Subgroup analysis showed improved SE and similar SP (PPV, NPV) for high grade (HG) recurrence, with a SE of 74% and SP of 89% (39%, 97%). NBI cystoscopy does not necessarily provide additional benefit over standard WLC. However, the XBCM may provide better SE and a diagnostic advantage in instances of HG disease recurrence.

## 1. Introduction

Urothelial carcinoma of the bladder (BC) is one of the most common cancers worldwide. Cigarette smoking is the most important risk factor for the disease [1,2]. Approximately 75% of BC patients have non-muscle invasive tumors (NMIBC), while the remaining 25% are muscle invasive (MIBC). Untreated MIBC has a higher cancer-specific mortality (CSM; 41%) after five years compared to NMIBC patients (7%) [2,3]. Despite the lower CSM, NMIBC has a higher probability of disease recurrence (approximately 70%) within 5 years. Therefore, it is likely that the majority of patients diagnosed with BC have NMIBC that will progress to MIBC if left untreated, and this mandates frequent and reliable follow-up (FU) [4].

Following transurethral resection of bladder tumors (TURBT), which carries both diagnostic and therapeutic value, the gold standard for NMIBC surveillance includes white light cystoscopy (WLC), urine cytology, and upper-tract imaging via CT or MR urogram, intravenous or retrograde pyelography, or ureteroscopy based on the presence of low-, intermediate-, or high-risk disease, all of which may improve disease-specific survival [5]. However, NMIBC surveillance presents a number of challenges for both clinicians and patients alike. Namely, non-compliance with a strict surveillance regimen is more likely to lead to unfavorable outcomes in terms of progression, recurrence, and metastasis [6]. One key compliance hurdle is that the frequency of strict surveillance is a costly endeavor. For this reason, higher-risk disease is more expensive than screening for lower-risk disease, although the greatest cost is ultimately due to disease progression [7]. A second challenge is that cytology requires the expertise of an experienced pathologist, preference a genitourinary pathologist, and has limited sensitivity (SE) in instances of low-grade (LG) disease [8]. In addition, interpretation can vary between pathologists. Third, WLC is less useful for detecting flat, sessile lesions, that, if missed, may progress to muscle-invasive and/or metastatic disease [2]. Finally, although TURBT is diagnostically and therapeutically effective, it is usually used with concurrent WLC and carries an increased risk of incomplete resection, which in turn raises the risk of disease progression or metastasis [9].

Several complementary technologies have emerged to help mitigate these limitations. One visual imaging option utilizes hexaminolevulinate (HAL)-fluorescence cystoscopy (also termed “blue-light” cystoscopy, or BLC), which relies on the preoperative intravesical instillation of HAL, a small-molecule ester preferentially taken up by tumor cells and re-expressed on the mucosa as a photoactive porphyrin. With BLC, normal tissue appears dark blue or purple, while cancerous tissue fluoresces bright pink. In conjunction with WLC, BLC may improve the diagnostic accuracy of cystoscopy and facilitate a more complete tumor resection, thereby reducing the rate of tumor recurrence in NMIBC disease [10]. However, BLC is more expensive and is logistically complex compared to WLC [11]. Moreover, BLC results can be confounded by acute and chronic inflammation, such as the state of the bladder during or after a urinary tract infection(s) or in instances of traumatic penetration of the cystoscope into the bladder.

Other promising technologies include the milieu of novel markers in the urine, such as enzyme immunoassays (NMP22©; Alere, Waltham, MA, USA), H-related proteins (BTA STAT^®^ and BTA TRAK©; Polymedico, Cortlandt, NY, USA), immunohistochemical assays (ImmunoCyt/uCyt+©; Scimedex, Dover, NJ, USA), fluorescence in situ hybridization (UroVysion©; Abbott, Chicago, IL, USA), multiplex immunoassays (Oncuria©; Nonagen Bioscience, Jacksonville, FL, USA), oncoproteins (Uro17©; Accupath, Plainview, NY, USA), and mRNA (Cxbladder©; Pacific Edge, Hummelstown, PA, USA) [12,13,14,15,16,17,18].

In this study, we had the opportunity to simultaneously investigate the impact of two other emerging technologies on BC surveillance–Narrow Band Imaging© cystoscopy (NBI; Olympus, Tokyo, Japan), and the XPERT Bladder Cancer Monitor© (XBCM; Cepheid, Sunnyvale, CA, USA). NBI is based on a light spectrum involving two different wavelengths (blue (415 nm) and green (540 nm)) that reduce red light, thereby improving the visibility of hyper-vascularized areas often seen near tumors. Some have stated that NBI provides better visibility and NMIBC detection rates compared to WLC and BLC, and that the system is easier to use and requires no additional reagents [19,20].

XBCM is an assay for voided urine that detects five mRNA sequences (ABL1, CRH, IGF2, UPK1B, ANXA10) [21,22]. The fully automated, integrated procedure has an average turnaround time of approximately two minutes, provides a result within 90 min, and has been validated in several prospective studies across multiple centers, demonstrating an improved SE and negative predictive value (NPV) compared to urine cytology, which was less effective for both parameters in both low- and intermediate-grade disease [23,24,25,26,27].

Here, we report the findings of our prospective single-center study aimed at (a) evaluating the clinical performance characteristics of XBCM and NBI compared to WLC, urine cytology, and histopathology via TURBT, and (b) using the linear discriminant analysis (LDA) of XBCM to identify different thresholds to potentially improve upon the SE of the test.

## 2. Materials and Methods

### 2.1. Study Design and Participants

This was a prospective, double-blind, single-center study conducted at a tertiary referral center in Switzerland. We enrolled men and women over 18 years of age who had already been diagnosed with ≤T1 NMIBC and were enrolled and followed-up for routine BC surveillance between 1 January 2018 and 31 March 2020. We excluded those who (a) were treated with intravesical BCG within 6 weeks before XBCM or NBI cystoscopic analysis, (b) underwent TURBT less than 12 weeks before XBCM or NBI cystoscopic analysis, (c) underwent concurrent treatment for metastatic disease of any origin (to avoid the potential of confounder mRNAs in voided urine), and/or (d) patients with ≥T2 BC. All urologists and patients were blinded to the XBCM results. All participants provided written informed consent.

An experienced cytopathologist and genitourinary pathologist experienced in molecular pathology analyzed the cytology specimens, tissue samples from bladder biopsies and/or TURBT, and the XBCM results. Five attending urologists at the institution were actively involved in cystoscopy, urine cytology, and tumor tissue specimen collection. As is standard clinical practice, the entire study patient population was independently divided among the five attending physicians. All senior physicians performed approximately the same number of cystoscopies during the study period. Both pathologists were blinded to patient identifiers and clinical status. The urology attendings and the urology resident were blinded to XBCM results.

### 2.2. XBCM and NBI Cystoscopy Logistics

In addition to the standard institutional NMIBC follow-up scheme (consisting of WLC, urine cytology, and renal ultrasound), all study patients were also examined by XBCM and NBI cystoscopy. The XBCM sample was obtained from voided midstream urine samples that were collected prior to same-day WLC and NBI cystoscopies. The assay was performed in full compliance with the manufacturer’s instructions and guidelines. The XBCM assay was considered positive for NMIBC disease recurrence if the LDA value was equal to or greater than 0.5, while LDAs less than 0.5 were considered negative for disease recurrence.

Urine cytology was collected during WLC and after gentle irrigation of the bladder with 150 mL normal saline. This cleared the bladder of pre-existing debris and contamination while ensuring standardized cytology sample collection across the study population. Cytology was considered to be positive if severe urothelial nuclear atypia was noted.

In all patients, the examining urologist graded the bladder mucosa on WLC as suspicious (i.e., visually positive), not suspicious (i.e., visually negative), or indeterminate (e.g., focally erythematous sans other characteristics or finely papillary). Many of those with suspicious features had lesions large and/or numerous enough to warrant bladder biopsy or TURBT, which was performed to obtain a tissue sample for histopathologic diagnosis (H) and analysis (WLC + H). The remaining patients with suspicious features were found to have visually small, low-grade, papillary lesions that warranted only continued visual surveillance (WLC) without tissue resection in accordance with published guidelines [5,28,29]. The tumors were histopathologically classified as low- or high-grade.

### 2.3. Handling Follow-Up Data

As this study was conducted in a real-world setting, we also examined more ambiguous situations as reported by the treating urologist and relied on the nature of follow-up examinations to further stratify a subset of the cohort. In patients with (a) suspicious WLCs without tissue collection or (b) indeterminate WLCs, follow-up was further divided into “simple” (FUS) or “total” (FUT), defined below, with short- or long-term follow-ups in both cases. This further classification was performed to enhance the accuracy of our results and reduce possible bias.

FUS: In instances of suspicious WLC without immediate tissue sampling via bladder biopsy or TURBT, we investigated the incidence of follow-up bladder biopsy or TURBT of the same lesion later in the patient’s follow-up schedule. If the patient underwent subsequent sampling of the lesion, the histopathologic findings were evaluated at this later time point to ascertain the patient’s BC status. If the patient was found to have BC, they were classified as having a “positive” cancer status; similarly, if TURBT showed a benign finding, they were classified as having “negative” cancer status. Cases of indeterminate WLC were handled similarly in that if an area of concern was eventually sampled and found to be BC, the patient’s cancer status was deemed “positive”. If further follow-up was uneventful with subsequent non-suspicious WLC, the patient’s cancer status was deemed “negative”.

FUT: Due to the blinded design of this study, it is possible and likely that we encountered cases with a positive XBCM test result but otherwise non-suspicious WLC. In these cases, we did not perform random bladder tissue sampling and thus, these patients were noted to have “missing” histology. The concept of FUT is interesting to us, because with respect to the reference definitions mentioned (WLC, WLC + H, FUS), a patient with a positive XBCM could be interpreted to have either a false positive (no actual BC, spurious XBCM result) or true positive (positive XBCM result and a lesion missed on WLC) result. Thus, to us, the only feasible way to increase the certainty of a true positive was to assess the follow-up progression of such cases by defining FUT as follows: if a patient’s subsequent WLC or histology was positive within 3 months of a positive XBCM, the patient’s cancer status was deemed to be “positive”. If follow-up WLC or histology was not suspicious for disease, the patient’s cancer status was deemed “negative”. In the latter “negative” cases, FUT allowed us to gain confidence with a truly negative cancer status and classify a suspect XBCM positive result as “falsely positive” rather than the alternative.

### 2.4. Data Collection and Statistical Analysis

Assuming a SE difference of 25% between cytology and XBCM and recurrent BC prevalence of 30%, 214 examinations were required to achieve 81% significance. Data were recorded and stored in a secured Excel spreadsheet (Microsoft Corp., Redmond, WA, USA). Descriptive statistics were presented as counts and frequencies for categorical data. Medians (with interquartile ranges) were presented for metric variables. Overall *p*-values corresponded to Kruskall-Wallis tests, chi-squared tests, or Fisher exact tests if the expected frequencies were below five. *p* < 0.05 was considered significant. Test validity measures (SE, specificity (SP), NPV, and positive predictive value (PPV)) were computed using 95% binomial confidence intervals. Receiver operating characteristic (ROC) curves were presented with their corresponding areas under the curves (AUC) and 95% confidence intervals. All evaluations were performed using the statistical software R (version 3.6.1).

### 2.5. Ethics and Institutional Approval

All patients included in the study provided written informed consent. The study was approved by the Swiss Ethics Committee (approval # EKNZ 2017-02061, date of approval: 12 January 2018).

## 3. Results

### 3.1. Description of the Cohort

Between January 2018 and March 2020, 301 WLCs were performed in 139 patients (median age 66; 105 males, 34 females) with a history of NMIBC. Of the 301 WLCs, 116 (38.5%) initially had low-grade disease while 153 (50.8%) demonstrated high-grade urothelial carcinoma. 31 patients (10.2%) had an unknown BC status (Table 1). 11 patients presented with hematuria at initial follow-up, 93 did not, and 32 had an unknown or undocumented hematuria status. 53 patients were current or former smokers, 23 were nonsmokers, and 60 had an unknown or undocumented smoking status. 20 patients were initially diagnosed with pT1 disease, 111 with pTa, and 5 with pTis. The carcinoma was high-grade in 63 patients, low-grade in 62, and unknown in 11. With regards to EORTC risk classification, 6 patients were classified as very-high risk, 41 as high-risk, 31 as intermediate-risk, and 58 as low-risk. At the time of enrollment, 50 patients had received or were in the process of receiving intravesical BCG, 85 did not receive BCG, and the BCG status was unknown in one patient.

### 3.2. Exclusions

Several patients were excluded from the study and we wish to briefly comment on these. One patient was excluded because he had metastatic pancreatic cancer involving the right kidney. He died shortly thereafter. Another had metastatic sarcomatoid cancer and underwent palliative chemotherapy. A third was excluded due to metastatic lung cancer and also underwent palliative chemotherapy.

Three patients were excluded due to misclassification: two were originally diagnosed with T2/3 tumors and had already received radio-chemotherapy, and one underwent XBCM and cystoscopy due to acute and severe indolent macrohematuria with no history of previous BC; he was not considered to be a follow-up case.

Three highly frail patients were noted to have easily identifiable papillary tumors on WLC. These tumors grew slowly per follow-up WLCs. Given the slow growth kinetics and patient frailty, the patients and urologists mutually agreed against performing TURBTs and these patients were ultimately excluded as well. One additional patient was noted to have BC on WLC, but died in an accident shortly before a planned TURBT; this patient was also excluded.

### 3.3. Description of Patient Subgroups

Our results are presented according to one the four reference definitions (WLC, WLC + H, FUS, and FUT). 301 WLCs (52 positive (17%), 220 negative, 29 unclear), 299 urine cytologies (21 positive, 278 negative), and 300 XBCM tests (62 positive, 236 negative, 2 invalid) were performed.

TURBT was performed in 44 (15%) cases with histopathologically proven BC in 33 (11%) cases. In 15 cases with a positive WLC, TURBT was not performed (11 had very small lesions, 3 patients opted against TURBT, and 11 patient died). In 29 cases, WLC was unclear/inconclusive; thereof these patients underwent TURBT, with negative resulting pathology.

For the FUS subgroup analysis, we assessed the follow-up (median FU 17 months) of cases with initial positive or equivocal WLC without initial TURBT. According to the FUS definition, the reference status was changed from positive to negative in 5 cases, from unclear to negative in 20 cases, and from unclear to positive in 6 cases. Five patients were excluded for the above reasons during the FU period, 12 TURB have been performed.

In those undergoing further evaluation of initially positive XBCM results in the absence of a positive WLC (i.e., FUT subgroup analysis), the same 5 patients were excluded after FUS. In 9 of 27 cases (33.3%), follow-up WLC was positive (median follow-up was 17 months), and the reference status for FUT calculation was then changed to positive.

### 3.4. Urine Cytology

18 cases demonstrated positive urine cytologies while 251 were negative; corresponding WLCs were positive in 50 cases (Table 2). The SE and SP for cytology in relation to WLC was 28% and 98%, respectively, with a PPV of 86% and NPV of 79%. We then correlated the cytologic and histopathologic results of TURBT with WLC (WLC + H), which demonstrated suspicious cytology in 19, negative cytology in 252, and positive histopathology in 44 cases. The cytologic SE and SP in WLC + H was 27% and 97%, respectively, with a PPV and NPV of 63% and 87%, respectively. For the reference definition FUS, SE and SP were 26% and 97%, respectively, and PPV and NPV of 60% and 88%, respectively. For the reference definition FUT, SE and SP were 27% and 97%, respectively, with a PPV and NPV of 63% and 87%, respectively.

### 3.5. XBCM Voided Urine Assay

With WLC as the reference definition, 45 (16.8%) of the 268 cases demonstrated a positive XBCM (223 negative) (Table 3). Yet, WLC was suspicious in only 50 cases. In this context, XBCM had a SE and SP of 48% and 90% (PPV of 53%, NPV of 88% and AUC of 0.764 (0.687–0.841)). Using WLC + H as the reference, XBCM had a SE and SP of 50% and 89%, and a PPV and NPV of 47% and 90% (AUC 0.759 (0.676–0.842)). Using the FUS and FUT as reference definitions, SEs were 57% and 87%, respectively, and SPs 58% and 89%, respectively (PPV 46% (FUS) and 51% (FUT); NPV 92% (FUS) and 92% (FUT); AUC 0.779 (0.698–0.859; FUS) and 0.794 (0.716–0.871; FUT)).

### 3.6. Boxplot Calculations 

**Figure 1:**
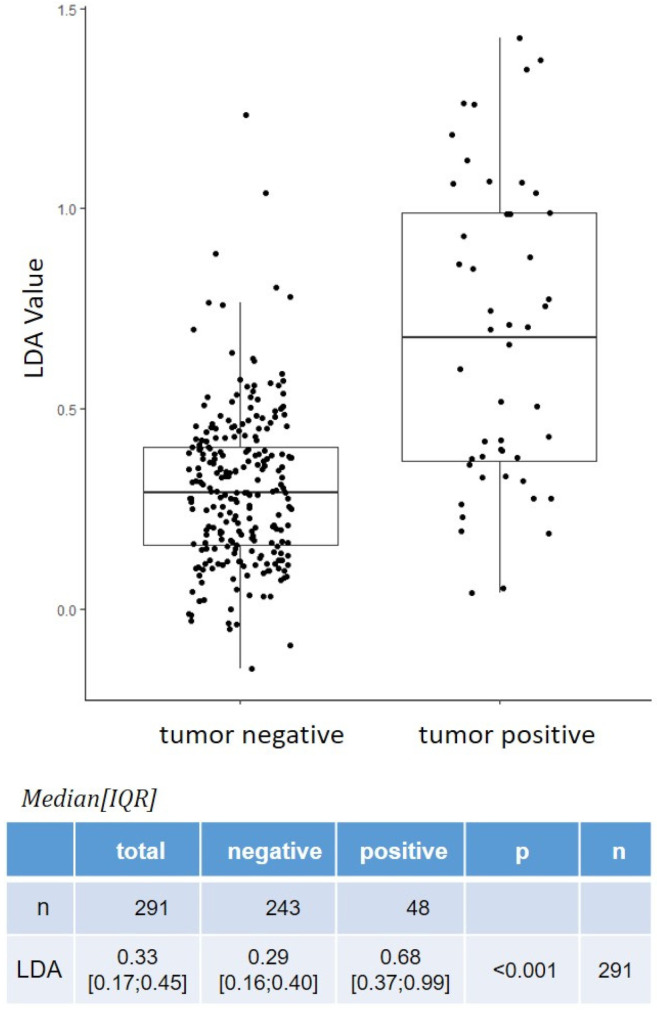
Boxplot of XBCM LDA values for tumor negative and tumor positive cases.

As mentioned earlier, the binary positive/negative XBCM result (i.e., positive or negative) is based on a pre-defined cut-off linear discriminant analysis (LDA) value. In a boxplot diagram (Figure 1), the LDA values (using FUT as a reference) for histopathologically tumor-negative and tumor-positive cases were plotted. Clustering was seen with the tumor-negative LDA values, whereas increased variability was seen with tumor-positive cases. The median interquartile range (IQR) of LDA values for all cases (*n* = 291) was 0.33 (0.17; 0.45). For tumor-negative (*n* = 243) and tumor-positive (*n* = 48) cases, the median IQR was 0.29 (0.16; 0.40) and 0.68 (0.37; 0.99), respectively (*p* < 0.001).

### 3.7. Variation between Low- and High-Grade Disease

**Figure 2:**
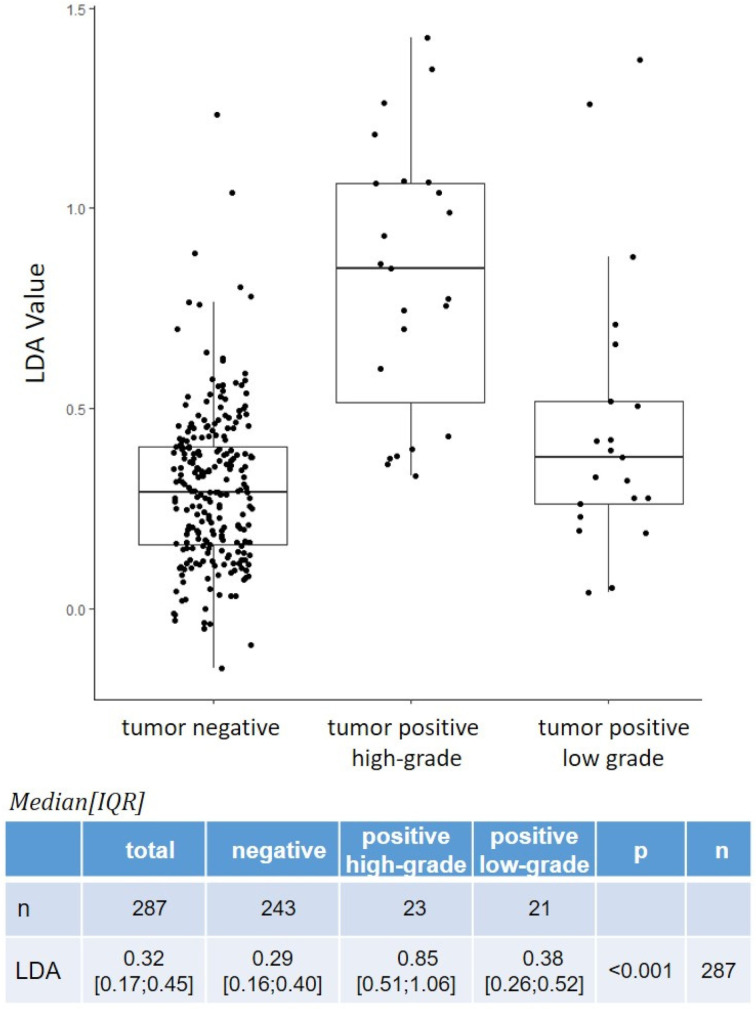
Boxplot of XBCM LDA values for tumor positive high-grade and low-grade cases.

Considering the subgroups of low-grade (LG, *n* = 21) and high-grade (HG, *n* = 23) disease recurrences, the LG SE and SP were 33% and 74%, while the HG SE and SP were 89% and 89% (LG PPV and NPV were 21% and 94%, respectively, while HG PPV and NPV were 39% and 97%, respectively). The same boxplot calculations were performed for both recurrence subtypes as well as negative (benign) tumors (Figure 2). LG tumors tended to have lower LDA values than HG recurrences. The median IQR of LDA values for all BC recurrences was 0.56 (0.35; 0.95). For LG (*n* = 21) and HG (*n* = 23) tumors, the median IQR was 0.38 (0.26; 0.52) and 0.85 (0.51; 1.06), respectively (*p* < 0.001). For the same cases compared to negative cases, the median IQR for negative tumors was 0.29 (0.16; 0.40) (*p* < 0.001).

### 3.8. Thresholds

**Figure 3:**
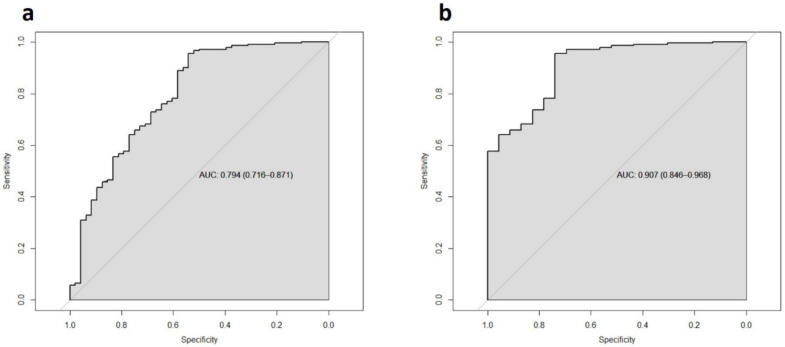
Receiver operating characteristic (ROC) curves for sensitivity (SE) and specificity (SP) of XBCM according to the reference definition follow-up total (FUT): tumor overall (**a**), high-grade tumor (**b**).

The XBCM binary result is entirely dependent on the LDA threshold (0.5) reported by Cepheid. However, we wanted to determine if changing this threshold could improve the informative value of the test. ROC curves were generated for XBCM using the FUT definition as a reference, with an AUC of 0.794 (0.716–0.871) (Figure 3a).

After calculating thresholds for SEs between 85% and 95%, a selection of SEs was made. For example, a LDA threshold value of 0.76 resulted in a SE and SP of 98% and 40%, respectively. A threshold of 0.85 resulted in a SE and SP of 99% and 35%, while a threshold of 0.90 resulted in a SE of 99% and SP of 31%. Obviously, the SP decreases as the threshold is further increased, which does not add further value to the test from a clinical point of view (for one more example, an LDA threshold of 1.0 yielded a SE of 99% and SP of 23%).

The same calculations were performed for the HG tumor subset, with an AUC of 0.907 (0.846–0.968) (Figure 3b). At an LDA threshold of 0.40, the SE was 75% and SP was 78%. At an LDA threshold of 0.45, SE and SP were 81% and 74%, respectively. At an LDA of 0.5 (i.e., manufacturer specification), SE and SP were 88% and 74%; at 0.6, 95% and 70%; at 0.7, 97% and 70%; and at 0.8, 98% and 0.52% (Table 4).

### 3.9. Narrow Band Imaging Cystoscopy

NBI cystoscopy was performed immediately after WLC in 289 of 301 cases (it was not performed or not documented in 12 cases). In all 289 cases, NBI cystoscopy findings matched WLC findings (100%). Because there was absolutely no difference between the two, further statistical analysis was considered unnecessary. NBI did not add any value or additional information to BC follow-up in our cohort.

## 4. Discussion

In this study, we investigated the performance of urine cytology, the XBCM voided urine assay, WLC, and NBI cystoscopy in 301 BC follow-up cases. The decision not to biopsy all suspicious lesions reflects the “real world” nature of our study cohort and follows a contemporary approach to patient follow-up that includes the active surveillance of unclear and very small lesions. To briefly summarize our results, in our cohort, cytology showed a high SP (97%) and moderate SE (27%), with PPV and NPV of 65% and 87%, respectively. Compared to cytology, XBCM demonstrated a higher SE (58%) but a lower SP (89%), resulting in a better NPV (92% for XBCM vs. 87% for cytology) but lower PPV (51% for XBCM vs. 65% for cytology). Moreover, the SE for XBCM was better for HG (74%) than LG (33%) recurrences, with identical SPs (89%) between the two groups.

The binary test-result (positive vs. negative) of XBCM is based on a linear discriminant analysis (LDA) cut-off value set by the manufacturer (LDA = 0.5). However, our ROC curve calculations demonstrated that adjusting the threshold value could improve test performance. For example, a threshold of 0.52 yielded a SE of 90% and a PPV of 88%. However, increasing the SE further resulted in a lower SP and NPV (36% and 40%, respectively).

We question whether mRNA-based tests, such as XBCM, should provide only binary results. For instance, in routine follow-up, clinical interpretation and shared decision making often depend on concomitant factors and the overall clinical situation. This study’s design does not answer this question directly; not all patients with equivocal WLCs received TURBT, as active surveillance is an established option at our institution. However, in the real world setting of our study, the question of whether or not to perform TURBTs in instances of equivocal WLC, XBCM, or urine cytology is one that we encounter regularly. Therefore, urologists should be able to decide if SP or SE is more important in each unique situation. We suggest that XBCM should augment its binary result at a given threshold with the associated SEs and SPs at other thresholds (such as those shown in Table 4)

### 4.1. Comparison with Other Studies Using XBCM

Elsawy and colleagues published a recent paper examining the performance of XBCM in patients undergoing active surveillance for NMIBC [23]. Their study included 168 patients, 10% of whom had biopsy-proven NMIBC recurrence. XBCM was found to have a SE of 74% and NPV of 96%. All their HG recurrence cases had positive XBCM results, consistent with our own finding that XBCM is particularly sensitive for HG carcinomas. In their cohort, urine cytology, with its SE of 47% and NPV of 93%, did not perform as well as XBCM [23]. However, the SP of XBCM (79%) was lower than that of urine cytology (85%). They concluded that XBCM performed better than urine cytology and could be helpful in excluding and predicting BC recurrence.

Other previous studies found a NPV of 89% for HG recurrences, while the SE for LG tumors was 71% compared with 21% for urine cytology [26]. This result was validated by Pichler et al., who found a SE of 77% for LG tumors [21]. In a multicenter study, Valenberg et al. evaluated the performance of XBCM in patients with hematuria and without a prior BC diagnosis [26]. In that study, XBCM demonstrated a SE of 78% and SP of 90% for HG tumors. The overall SP was 84% and NPV was 98%. D’Elia and colleagues studied 230 patients enrolled in active surveillance for NMIBC and found that XBCM had a higher SE and SP than urine cytology (SE: 46% vs. 11%, SP: 97% vs. 77%) [25]. bei Hurle and colleagues examined 106 patients who were monitored after a NMIBC diagnosis and used an even lower LDA cut-off value than D’Elia et al. (LDA of 0.4 vs. the manufacturer specification of 0.5) [24]. They concluded that using a lower cut-off value avoided 22% of WLCs by missing only 9% of cancer recurrences in total (and 0% with HG lesions), while SE and SP were 30% and 94%, respectively (PPV: 91%, NPV: 40%). When an LDA of 0.5 value was chosen as the cut-off, a SE and SP of 30% and 90% were reported (PPV: 82%, NPV: 47%).

To compare and contrast, our study demonstrated that urine cytology showed a SE and SP of 27% and 97% (PPV: 65%; NPV 87%), respectively, whereas with XPERT©, the SE and SP were 58% and 89% (PPV: 51%; NPV: 92%), respectively. Thus, compared to other studies, XBCM in our hands and cohort showed a lower SE and NPV but higher SP. Like other groups, the HG subgroup of our study cohort demonstrated better SE but similar SP, PPV, and NPV for HG disease recurrence versus LG recurrence, with a SE of 74% and SP of 89% (PPV: 39%; NPV: 97%) vs. SE of 33% and SP of 89% (PPV: 21%; NPV: 94%), respectively.

### 4.2. Comparison with Other Tests

There have been many attempts to improve BC detection using urine biomarkers. The number of BC urine biomarker tests has increased dramatically in recent years, and despite the overall goals of reducing invasive testing and improving diagnostic accuracy, various studies have yet to find a suitable replacement for WLC and urine cytology as the gold standard [30,31,32]. One of the first urine cytology alternatives was NMP22©, first described by Zippe and colleagues in 1999 as a solitary urine marker for the detection of bladder transitional cell carcinoma. NMP22 harbored a SE of 100%, SP of 85%, and NPV of 100% compared urine cytology, which demonstrated a SE and SP of 33% and 100%, respectively [17]. Another alternative is BTA STAT©, a qualitative point-of-care assay with a SE and SP of 68.7% (53–89%) and 73.7% (54–93%), respectively, and several studies have shown wide variability for both of these values, while BTA TRAK is a quantitative enzyme-linked immunosorbent assay (ELISA) [30]. UroVysion©, a fluorescence in situ hybridization assay that identifies aneuploidy for chromosomes 3, 7, 17, and loss of the 9p21 locus demonstrated a SE and SP of 39.1% and 89.7%, respectively. Compared to urine cytology, which showed a SE and SP of 40.6% and 89.7%, respectively, this suggested that the new assay offered limited or no additional benefit [16,33]. Konety and colleagues evaluated NPV in a retrospective study using pooled data for Cxbladder©, an assay measuring MDK, HOXA13, CDC2, IGFBP5, and CXCR2 mRNA levels. Cxbladder showed an NPV of 97% compared to urine cytology (93%), and was deemed helpful in cases of negative WLC and suspicious urine cytology for identifying patients who merited further workup or enhanced surveillance [34]. Uro17© is a test that measures levels of the oncoprotein K17 in voided urine cytology samples. Initial testing in patients suspected of having BC (48 of 71 patients with BC including carcinoma in situ) resulted in a SE of 100% and SP of 92% (PPV and NPV of 97.5% and 100%, respectively). Oncuria© is a multiplex immunoassay that detects the biomarkers A1AT, APOE, ANG, CA9, IL8, MMP9, MMP10, PAI1, SDC1, and VEGFA, and also showed promising results in 46 de novo BC patients compared with non-cancer controls, with a reported SE and SP of 0.93 each and PPV and NPV of 65% and 99%, respectively [14]. However, both Uro17© and Oncuria© have not yet been evaluated in the follow-up/surveillance setting.

### 4.3. NBI Cystoscopy

In all of our cases, NBI cystoscopy showed exactly the same results as WLC. We did not detect more tumors with NBI cystoscopy. Therefore, NBI cystoscopy did not provide any further information that would have changed our clinical decision making or management. Some of the early studies that assessed NBI cystoscopy did demonstrate a better BC detection rate compared to WLC [19]. For instance, Li and colleagues performed a meta-analysis that looked at over 1000 patients and showed a random effect estimate of 17% of the patients in favor of NBI [20]. However, the most recent and potentially most informative study evaluating NBI cystoscopy, particularly in NMIBC follow-up, was designed as a randomized controlled trial and divided patients into two groups of 300 patients each (group 1: WLC only; group 2: WLC + NBI cystoscopy) [35]. This study’s results were congruent with ours, in that NBI cystoscopy conferred no significant additional benefit over WLC in NMIBC follow-up.

### 4.4. Outlook

The overarching goal with introducing new BC diagnostic tools into routine clinical practice is to positively change the way we perform follow-ups. Perhaps we will one day be able to reduce the frequency of or even eliminate cystoscopy altogether. To accomplish such a tradeoff, emerging assays and algorithms need to demonstrate increased safety and reliability. To this end, if the XBCM is available at a medical facility, it can provide rapid and accurate results. However, if it not available, building the XBCM institutional ecosystem can have a high upfront cost, and the issue of reimbursement has yet to be clarified in many healthcare systems and countries. An interesting and noteworthy feature of the XBCM platform is that the technology can be adapted on the fly by adding other mRNA markers using the exact same hardware. This will likely streamline and accelerate development processes and potentially enhance XBCM accuracy over time as new biological and genomic data are unearthed. Lastly, aside from screening and follow-up, one of the most interesting roles for urine-based molecular testing is in the context of systemic or intravesical instillation therapies, where such tests could play a role in stratifying treatment risks and response [36]. Further validation of XBCM and other molecular tests is required in such settings.

### 4.5. Study Strengths and Limitations

The strengths of this study are primarily three-fold. First, it was performed prospectively in a blinded fashion at a single center. Second, it evaluated not only XBCM but also NBI cystoscopy for routine follow-up in a real-world setting. Third, longitudinal follow-up data was included.

It is also important to recognize and discuss this study’s limitations. First, the sample size of 300 cases is small. To obtain more robust statistical results, a larger cohort would be desirable. However, with the number of cases exceeding 300, our study is comparable to the other reference publications on this topic. Second, not all patients classified as cystoscopically positive received (immediate) TURBT. Again, this was due to the real-world nature of the study and the clinical practice patterns of our institution. Our clinical decision making was in line with the evidence-based recommendation that active surveillance may be an option for some patients, resulting in patients and/or treating physicians being able to decide on whether or not it was appropriate to pursue active surveillance for small papillary tumors [37].

## 5. Conclusions

We conclude that WLC still remains the gold standard for follow-up of potentially recurrent NMIBC. In our hands, NBI cystoscopy did not provide an additional benefit over standard WLC for routine follow-up. Moreover, the XBCM voided urine assay platform may provide better sensitivity and a diagnostic advantage in instances of high-grade NMIBC recurrence. Given the technological possibility of augmenting such a platform over time, XBCM remains a promising tool for the future.

## Figures and Tables

**Table 1:** Cohort Demographics.

Total Patients	139	
Median age (years)	66	
Males	105	
Females	34	
Exclusions		
Metastatic disease (non-BC)	3	
Failed inclusion criteria		
Initially > pT1	2	
No prior BC	1	
Notable concurrent factors		
Prior BCG treatment	50	36%
Hematuria	11	8%
Smokers	53	38%
Initial T-stage		
pTis	5	4%
pTa	111	80%
pT1	20	14%
Initial grading		
High-grade	63	45%
Low-grade	62	44%
Unknown	11	8%
EORTC risk classification		
Very-high	6	4%
High	41	29%
Intermediate	31	22%
Low	58	42%

WLCs: white light cystoscopies; XBCM: Xpert© bladder cancer monitor; NBI: narrow. Band imaging©; TURBTs: trans-urethral resection of bladder tumors.

**Table 2:** Urine Cytology.

Analysis	WLC	WLC + H	FUS	FUT
tprev	0.1859	0.1624	0.1565	0.1644
SE	0.28	0.2727	0.2609	0.2708
SP	0.9817	0.9692	0.9677	0.9713
PPV	0.7778	0.6316	0.6	0.65
NPV	0.8566	0.873	0.8759	0.8713

Sensitivity (SE) and specificity (SP) of urine cytology according to the four reference definitions: white-light cystoscopy (WLC), white-light cystoscopy and histology (WLC + H), simple follow-up (FUS) and total follow-up (FUT). True prevalence (tprev).

**Table 3:** XBCM Voided Urine Assay.

Analysis	WLC	WLC + H	FUS	FUT
tprev	0.1866	0.163	0.157	0.1649
SE	0.48	0.5	0.5652	0.5833
SP	0.9037	0.8894	0.8745	0.8889
PPV	0.5333	0.4681	0.4561	0.5091
NPV	0.8834	0.9013	0.9153	0.9153

Sensitivity (SE) and specificity (SP) of XBCM according to the four reference definitions: white-light cystoscopy (WLC), white-light cystoscopy and histology (WLC + H), follow-up simple (FUS) and follow-up total (FUT). True prevalence (tprev).

**Table 4:** Calculated selection of sensitivity (SE) and specificity (SP) of XBCM in high grade cases using different LDA-thresholds.

LDA	SE	SP
0.80	0.98	0.52
0.70	0.97	0.69
0.60	0.95	0.70
0.50	0.88	0.73
0.45	0.81	0.74
0.40	0.75	0.78

## Data Availability

The data presented in this study are available on request from the corresponding author. The data are not publicly available due to privacy.

## References

[1] Ferlay J., Steliarova-Foucher E., Lortet-Tieulent J., Rosso S., Coebergh J.W.W., Comber H., Forman D., Bray F. (2013). Cancer incidence and mortality patterns in Europe: Estimates for 40 countries in 2012. Eur. J. Cancer.

[2] Burger M., Catto J.W.F., Dalbagni G., Grossman H.B., Herr H., Karakiewicz P., Kassouf W., Kiemeney L.A., La Vecchia C., Shariat S. (2013). Epidemiology and risk factors of urothelial bladder cancer. Eur. Urol..

[3] Martini A., Sfakianos J.P., Renström-Koskela L., Mortezavi A., Falagario U.G., Egevad L., Hosseini A., Mehrazin R., Galsky M.D., Steineck G. (2020). The natural history of untreated muscle-invasive bladder cancer. BJU Int..

[4] Hollenbeck B.K., Dunn R.L., Ye Z., Hollingsworth J.M., Skolarus T.A., Kim S.P., Montie J.E., Lee C.T., Wood D.P.J., Miller D.C. (2010). Delays in diagnosis and bladder cancer mortality. Cancer.

[5] Babjuk M., Burger M., Compérat E.M., Gontero P., Mostafid A.H., Palou J., van Rhijn B.W.G., Rouprêt M., Shariat S.F., Sylvester R. (2019). European Association of Urology Guidelines on Non-muscle-invasive Bladder Cancer (TaT1 and Carcinoma In Situ)—2019 Update. Eur. Urol..

[6] Abushamma F., Khayyat Z., Soroghle A., Zyoud H.S., Jaradat A., Akkawi M., Aburass H., Qaddumi I.K.K., Odeh R., Salameh H. (2021). The Impact of Non-Compliance to a Standardized Risk-Adjusted Protocol on Recurrence, Progression, and Mortality in Non-Muscle Invasive Bladder Cancer. Cancer Manag. Res..

[7] Mossanen M., Chu A., Smith A.B., Gore J.L. (2019). Inferring bladder cancer research prioritization from patient-generated online content. World J. Urol..

[8] Tilki D., Burger M., Dalbagni G., Grossman H.B., Hakenberg O.W., Palou J., Reich O., Rouprêt M., Shariat S.F., Zlotta A.R. (2011). Urine markers for detection and surveillance of non-muscle-invasive bladder cancer. Eur. Urol..

[9] Klän R., Loy V., Huland H. (1991). Residual tumor discovered in routine second transurethral resection in patients with stage T1 transitional cell carcinoma of the bladder. J. Urol..

[10] Stenzl A., Burger M., Fradet Y., Mynderse L.A., Soloway M.S., Witjes J.A., Kriegmair M., Karl A., Shen Y., Grossman H.B. (2010). Hexaminolevulinate guided fluorescence cystoscopy reduces recurrence in patients with nonmuscle invasive bladder cancer. J. Urol..

[11] Klaassen Z., Li K., Kassouf W., Black P.C., Dragomir A., Kulkarni G.S. (2017). Contemporary cost-consequence analysis of blue light cystoscopy with hexaminolevulinate in non-muscle-invasive bladder cancer. Can. Urol. Assoc. J..

[12] Droller M.J. (1998). Improved detection of recurrent bladder cancer using the bard BTA stat test. J. Urol..

[13] Toma M.I., Friedrich M.G., Hautmann S.H., Jäkel K.T., Erbersdobler A., Hellstern A., Huland H. (2004). Comparison of the ImmunoCyt test and urinary cytology with other urine tests in the detection and surveillance of bladder cancer. World J. Urol..

[14] Hirasawa Y., Pagano I., Chen R., Sun Y., Dai Y., Gupta A., Tikhonenkov S., Goodison S., Rosser C.J., Furuya H. (2021). Diagnostic performance of OncuriaTM, a urinalysis test for bladder cancer. J. Transl. Med..

[15] Darling D., Luxmanan C., O’Sullivan P., Lough T., Suttie J. (2017). Clinical Utility of Cxbladder for the Diagnosis of Urothelial Carcinoma. Adv. Ther..

[16] Halling K.C., Kipp B.R. (2008). Bladder cancer detection using FISH (UroVysion assay). Adv. Anat. Pathol..

[17] Zippe C., Pandrangi L., Agarwal A. (1999). NMP22 is a sensitive, cost-effective test in patients at risk for bladder cancer. J. Urol..

[18] Chang S.S., Boorjian S.A., Chou R., Clark P.E., Daneshmand S., Konety B.R., Pruthi R., Quale D.Z., Ritch C.R., Seigne J.D. (2016). Diagnosis and Treatment of Non-Muscle Invasive Bladder Cancer: AUA/SUO Guideline. J. Urol..

[19] Herr H.W., Donat S.M. (2008). A comparison of white-light cystoscopy and narrow-band imaging cystoscopy to detect bladder tumour recurrences. BJU Int..

[20] Li K., Lin T., Fan X., Duan Y., Huang J. (2013). Diagnosis of narrow-band imaging in non-muscle-invasive bladder cancer: A systematic review and meta-analysis. Int. J. Urol. Off. J. Jpn. Urol. Assoc..

[21] Pichler R., Fritz J., Tulchiner G., Klinglmair G., Soleiman A., Horninger W., Klocker H., Heidegger I. (2018). Increased accuracy of a novel mRNA-based urine test for bladder cancer surveillance. BJU Int..

[22] Dobbs R.W., Abern M.R. (2018). A novel bladder cancer urinary biomarker: Can it go where no marker has gone before?. Transl. Androl. Urol..

[23] Elsawy A.A., Awadalla A., Elsayed A., Abdullateef M., Abol-Enein H. (2021). Prospective Validation of Clinical Usefulness of a Novel mRNA-based Urine Test (Xpert^®^ Bladder Cancer Monitor) for surveillance in Non Muscle Invasive Bladder Cancer. Urol. Oncol..

[24] Hurle R., Casale P., Saita A., Colombo P., Elefante G.M., Lughezzani G., Fasulo V., Paciotti M., Domanico L., Bevilacqua G. (2020). Clinical performance of Xpert Bladder Cancer (BC) Monitor, a mRNA-based urine test, in active surveillance (AS) patients with recurrent non-muscle-invasive bladder cancer (NMIBC): Results from the Bladder Cancer Italian Active Surveillance (BIAS) project. World J. Urol..

[25] Elia D.C., Pycha A., Folchini D.M., Mian C., Hanspeter E., Schwienbacher C., Vjaters E., Pycha A., Trenti E. (2019). Diagnostic predictive value of Xpert Bladder Cancer Monitor in the follow-up of patients affected by non-muscle invasive bladder cancer. J. Clin. Pathol..

[26] Van Valenberg F.J.P., Hiar A.M., Wallace E., Bridge J.A., Mayne D.J., Beqaj S., Sexton W.J., Lotan Y., Weizer A.Z., Jansz G.K. (2019). Prospective Validation of an mRNA-based Urine Test for Surveillance of Patients with Bladder Cancer. Eur. Urol..

[27] Wiener H.G., Vooijs G.P., van’t Hof-Grootenboer B. (1993). Accuracy of urinary cytology in the diagnosis of primary and recurrent bladder cancer. Acta Cytol..

[28] Hurle R., Colombo P., Lazzeri M., Lughezzani G., Buffi N.M., Saita A., Elefante G.M., Morenghi E., Forni G., Cardone P. (2018). Pathological Outcomes for Patients Who Failed to Remain under Active Surveillance for Low-risk Non-muscle-invasive Bladder Cancer: Update and Results from the Bladder Cancer Italian Active Surveillance Project. Eur. Urol. Oncol..

[29] Hurle R., Pasini L., Lazzeri M., Colombo P., Buffi N., Lughezzani G., Casale P., Morenghi E., Peschechera R., Zandegiacomo S. (2016). Active surveillance for low-risk non-muscle-invasive bladder cancer: Mid-term results from the Bladder cancer Italian Active Surveillance (BIAS) project. BJU Int..

[30] Serretta V., Pomara G., Rizzo I., Esposito E. (2000). Urinary BTA-stat, BTA-trak and NMP22 in surveillance after TUR of recurrent superficial transitional cell carcinoma of the bladder. Eur. Urol..

[31] Narayan V.M., Adejoro O., Schwartz I., Ziegelmann M., Elliott S., Konety B.R. (2018). The Prevalence and Impact of Urinary Marker Testing in Patients with Bladder Cancer. J. Urol..

[32] Sathianathen N.J., Butaney M., Weight C.J., Kumar R., Konety B.R. (2018). Urinary Biomarkers in the Evaluation of Primary Hematuria: A Systematic Review and Meta-Analysis. Bladder Cancer.

[33] Moonen P.M.J., Merkx G.F.M., Peelen P., Karthaus H.F.M., Smeets D.F.C.M., Witjes J.A. (2007). UroVysion compared with cytology and quantitative cytology in the surveillance of non-muscle-invasive bladder cancer. Eur. Urol..

[34] Konety B., Shore N., Kader A.K., Porten S., Daneshmand S., Lough T., Lotan Y. (2019). Evaluation of Cxbladder and Adjudication of Atypical Cytology and Equivocal Cystoscopy. Eur. Urol..

[35] Tschirdewahn S., Harke N.N., Hirner L., Stagge E., Hadaschik B., Eisenhardt A. (2020). Narrow-band imaging assisted cystoscopy in the follow-up of patients with transitional cell carcinoma of the bladder: A randomized study in comparison with white light cystoscopy. World J. Urol..

[36] Whitson J., Berry A., Carroll P., Konety B. (2009). A multicolour fluorescence in situ hybridization test predicts recurrence in patients with high-risk superficial bladder tumours undergoing intravesical therapy. BJU Int..

[37] Hernández V., Alvarez M., de la Peña E., Amaruch N., Martín M.D., de la Morena J.M., Gómez V., Llorente C. (2009). Safety of active surveillance program for recurrent nonmuscle-invasive bladder carcinoma. Urology.

